# Potentiating Effects of *Lactuca sativa* on Pentobarbital-Induced Sleep 

**Published:** 2013

**Authors:** Ahmad Ghorbani, Hassan Rakhshandeh, Hamid Reza Sadeghnia

**Affiliations:** a *Pharmacological Research Center of Medicinal Plants, School of Medicine, Mashhad University of Medical Sciences, Mashhad, Iran. *; b *Department of Pharmacology, School of Medicine, Mashhad University of Medical Sciences, Mashhad, Iran.*; c *Neurocognitive Research Center, School of Mashhad University of Medical Sciences, Mashhad, Iran. *

**Keywords:** *Lactuca sativa*, Pentobarbital, Sleep, PC12, Mice

## Abstract

Traditionally, *Lactuca sativa *(lettuce) has been recommended for its hypnotic property. The present study was planned to investigate sleep-prolonging effect of this plant**. **The hydro-alcoholic extract (HAE) of lettuce and its water fraction (WF), ethyl acetate fraction (EAF), and *n*-butanol fraction (NBF) were administrated (IP) to mice 30 min before the pentobarbital injection. Moreover, both *in-vivo *and *in-vitro *toxicity of the extracts were determined. The quality of HAE and NBF was also evaluated using HPLC fingerprint. The HAE prolonged the pentobarbital-induced sleep duration at dose of 400 mg/Kg. The NBF was the only fraction which could increase the sleep duration and decrease sleep latency. The effects of NBF were comparable to those of induced by diazepam. The LD_50_-value for HAE was found to be 4.8 g/Kg. No neurotoxic effect was observed either by HAE or by its fractions in cultured PC12 neuron-like cells. The results suggest that lettuce potentiates pentobarbital hypnosis without major toxic effect. The main component(s) responsible for this effect is most likely to be non-polar agent(s) which found in NBF of this plant.

## Introduction

Sleep is a physiologic recuperative state that can be disturbed by many factors such as illness, stress and noise. Chronic sleep disorder led to some health repercussions such as slower reactions, poor memorizing, emotional disturbances, and changes in the immune response (1, 2). Today, sleep disorders have a relatively high prevalence and are a growing public health problem. It is estimated that more than 27% of people worldwide suffer from sleep disorders with difficulty in initiating or maintaining sleep. In addition, it is expected that by the middle of the 21^st ^century, about 3-10% of all people will be chronic and frequent users of sleep medications (3-5). Currently, the most widely used medications for sleep disorders are the benzodiazepines. However, the clinical uses of benzodiazepines are accompanied with unpleasant side effects such as drug dependence, tolerance, rebound insomnia, amnesia, psychomotor impairment and potentiating of other central depressant drugs (6). Therefore, the search for new hypnotic agents with lesser side effects has continued. 

Medicinal plants have always been a good source to find new remedies for human health problems. *Lactuca sativa *(lettuce) an annual herb belonging to the *Compositae *family, has acquired a folk reputation due to its medicinal values. The leafy vegetable is commonly used fresh or in salad mixes (7). It has been reported to have a number of medicinal attributes including antioxidant, anti-inflammatory and analgesic activity (8, 9). Traditionally, lettuce has been suggested to have sedative-hypnotic property (7, 10, 11). However, there is no clear pharmacological evidence that this plant exerts a significant sedative-hypnotic effect or sleep-prolonging activity. The only report found in the literature was from the work of Gonzalex-lima *et al. *who demonstrated that the alcoholic extract of lettuce has sedative actions on behavior of toads (12).

Therefore, the present study was planned to evaluate the sleep-prolonging effect of lettuce hydro-alcoholic extract (HAE) and its fractions. Moreover, the possible neurotoxicity of the plant was assessed using PC12 neuron cells, a rat pheochromocytoma-derived cell line, to ensure that the effect accompanied with no negative impact on neurons.

## Experimental

*Drugs and chemicals*

Dimethyl sulfoxide (DMSO), pentobarbital sodium, penicillin-streptomycin and 3-(4,5-Dimethyl-2-thiazolyl)-2,5-Diphenyl-2H-tetrazolium bromide (MTT) were purchased from Sigma (Sigma, USA). Diazepam was obtained from Chemidarou Company (Iran). Tween 80 was from Merck (Merck, Germany). Dulbecco’s Modified Eagle’s Medium (DMEM) and fetal bovine serum (FBS) were bought from GIBCO (GIBCO, USA). For high performance liquid chromatography (HPLC), all solvents used were of HPLC-grade and purchased from Caledon (Caledon, Canada). Pentobarbital and diazepam were dissolved in saline to make a 3 mg/mL and 0.3 mg/mL solution, respectively.

*Preparation of extracts*

The fresh lettuce was purchased from local market in Mashhad, Iran. The plant sample was identified at the herbarium of school of Pharmacy (Mashhad University of Medical Sciences, Mashhad, Iran) and a voucher specimen (12596) has been deposited in this institute. The aerial parts of lettuce were dried, powdered and subjected to extraction with 70% ethanol in a Soxhlet apparatus for 48 h. The HAE extract was then dried on a water bath and the yield (37% w/w) dissolved in saline containing 1% (v/v) of Tween 80.

For preparation of fractions, a part of dried HAE was suspended in distilled water and transferred to a separator funnel. Using solvent-solvent extraction, it was sequentially fractionated with ethyl acetate and *n*-butanol. The ethyl acetate fraction (EAF) and *n*-butanol fraction (NBF) were separated to obtain water fraction (WF). The resulting fractions were dried on a water bath and working solutions made up in saline, saline containing 1% Tween, and 10% DMSO for WF, EAF and NBF, respectively (13).

*Animals*

Male albino mice weighting 22-32 g were used in each experiment. The animals were maintained at a controlled temperature with a 12 h light/dark cycle with free access to food and water. The study was conducted in accordance with ethical guidelines approved by the Animal Care Use Committee of Shiraz University of Medical Sciences. The animals were randomly divided into ten groups consisting of 8-12 mice each. In the first experiment, to determine if HAE has hypnotic effect, the following solutions were injected (IP) to six groups: saline as vehicle, diazepam (3 mg/Kg) as positive control, and HAE (50, 100, 200, 400 mg/Kg). In the second experiment, four groups of mice were treated (IP) with the following agents to determine the most effective fraction: WF, EAF, NBF and 10% DMSO (vehicle for NBF). The fractions were administered with dose of 200 mg/Kg according to our preliminary work.

*Sleep induction*

The sleep evaluation method was based on prolongation of pentobarbital-induced sleeping time (13-15). Briefly, the animals were given (IP) a single dose of the vehicles, diazepam, or the extracts. After 30 min, pentobarbital (30 mg/Kg, IP) was injected to induce sleep. The mice were considered asleep if stayed immobile and lost its righting reflex when positioned on its back. The time interval between pentobarbital injection and onset of sleep was recorded as sleep latency.

*LD*_50_ *determination*

Acute toxicity study was conducted by using the method described by Akhila *et al*. (16). Ten groups of two animals each were used. A wide range of doses of the HAE was tested (IP), starting from the lowest dose (50 mg/Kg), with increments of two. The treated animals were monitored for 24 h for mortality. The highest dose which did not killed any mice and the lowest dose which led to death of one mouse was noted. The geographic mean of these two doses was taken as the median lethal dose (LD_50_).

*Neurotoxicity assessment*

The PC12 cells were seeded in 96-well plates and cultured for 48 h in DMEM supplemented with 10% FBS, penicillin (100 units/mL) and streptomycin (100 μg/mL) at 37°C and 5% CO_2_. Then, the medium was changed to fresh one containing vehicle, HAE (200, 400, 800 and 1600 mg/L), or the fractions (800 mg/L). These concentrations were used based on the levels applied *in-vivo *and on the fact that extracellular volume of the rodent is approximately 25% of body weight (17). The cells were further incubated for 24 h. At the end of the treatment, the effect of extracts on cell proliferation was measured using MTT assay as previously described (13, 18). The assay was carried out in triplicate and repeated twice for confirmation. The level of cytotoxicity was expressed as the percent of surviving cells.

*Characterization of the extracts by HPLC*

The quality of HAE and NBF of lettuce was characterized by HPLC-UV fingerprint. It was carried out by a reverse-phase Waters C18 analytical column (250 × 4.6mm, 5 μm particle size), using an isocratic mobile phase of acetonitrile/water/H_3_PO_4 _(80:20:0.3% v/v) at a flow rate of 1 mL/min. The UV detector wavelength was set at 330 nm. The samples of HAE and NBF were dissolved in distilled water and acetonitrile, respectively, and passed through 0.45 μm membrane filter. Then, 20 μL of samples (400 μg/L) was injected to the HPLC column.

*Statistics*

All values were expressed as mean ± SEM. Statistical analysis was performed using one way analysis of variance (ANOVA) followed by Tamhane’s T2 post-hoc test. Differences were considered significant at p < 0.05.

## Results and Discussion

*Effects of lettuce on sleep*

**Figure 1:**
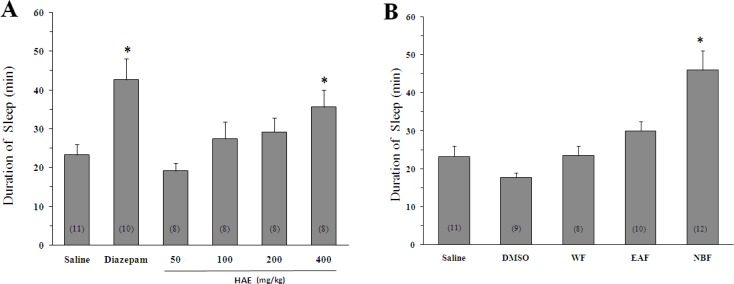
Effect of *Lactuca sativum *hydro-alcoholic extract (HAE) and its fractions on sleep duration in mice. (A) *p < 0.001 *vs *saline. (B) The animals were treated with 200 mg/Kg of HAE or its water fraction (WF), ethyl acetate fraction (EAF) and *n*-butanol fraction (NBF). *p < 0.01 *vs *DMSO. Data represent mean ± SEM of the numbers shown in parentheses.

Sleep duration in the animals receiving saline before pentobarbital injection was 23.3 ± 2.7 min (Figure 1). 

**Figure 2:**
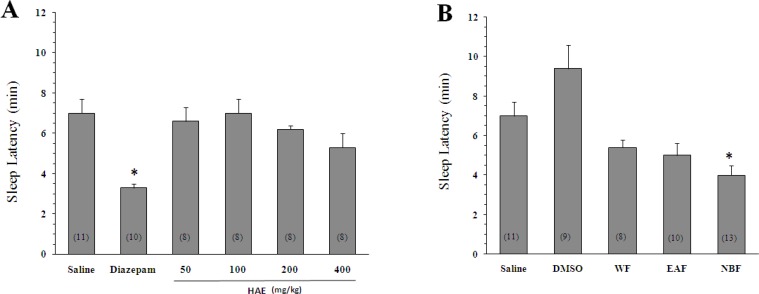
Effect of *Lactuca sativum *hydro-alcoholic extract (HAE) and its fractions on the sleep latency time in mice. (A) *p < 0.05 *vs *saline. (B) The animals were treated with 200 mg/Kg of HAE or its water fraction (WF), ethyl acetate fraction (EAF) and *n*-butanol fraction (NBF). *p < 0.01 *vs *DMSO. Data represent mean ± SEM of the numbers shown in parentheses.

As expected, diazepam could increase the pentobarbital-induced sleeping time (42.7 ± 5.5 min, p < 0.05 *vs *saline). Likewise, the HAE at doses of 400 mg/Kg significantly increased the sleep duration (35.7 ± 4.4 min, p < 0.05 *vs *saline). To obtain better insight into the nature of compounds responsible for the effect of HAE, three fractions were prepared: (1) The WF solubilizing the polar agents and water-soluble plant constituents (*e.g*. glycosides, quaternary alkaloids, tannins); (2) The EAF extracting compounds of intermediate polarity; (3) The NBF bearing non-polar agents like sterols, alkanes and some terpenoids (19, 21). As shown in Figures 1B and 2B, the NBF was the only fraction which could significantly prolong the sleep duration or decrease the sleep latency. When NBF was administrated, the sleep duration was increased to 46 ± 5.2 min *(*p < 0.01 *vs *DMSO) and the latency time was decreased from 9.4 ± 1.2 (vehicle) to 4 ± 0.5 min (p < 0.01). Neither the HAE doses (50, 100, 200 and 400 mg/Kg) nor the WF and EAF could cause a significant reduction in the sleep latency (Figure 2B). The fact that only NBF potentiates the sleep parameters indicates that non-polar agents are responsible for the effects of lettuce. Between the agents, existing evidences support the possible role for terpenoids: (1) Lactucin, a *sesquiterpene lactone *of *Lactuca *species, has been reported to have a sedative property in the spontaneous locomotor activity test (22); (2) Phytol, a diterpenoid isolated from the ethanolic fraction of lettuce was found to raise the levels of gamma-aminobutyric acid, a sleep-promoting neurotransmitter, in the brain (23). 

*Toxicity assessments*

The LD_50_-value for HAE was found to be 4.8 g/Kg. This value is so far from the effective dose of HAE, 400 mg/Kg. None of the HAE concentrations decreased proliferation of PC12 cells. In the presence of 200, 400, 800 and 1600 mg/L of the extract, surviving of the cells was 113 ± 2.4, 111 ± 2.3, 115 ± 1.8 and 112 ± 1.3%, respectively, as compared to untreated cells. Similarly, the HAE fractions exhibited no cytotoxicity. The level of surviving cells was 113 ± 2, 117 ± 1.4 and 102 ± 9 for WF, EAF and NBF, respectively. Therefore, the hypnotic effect of lettuce accompanied with no neurotoxicity, leading to further support of its safety. 

*Standard fingerprints*

**Figure 3:**
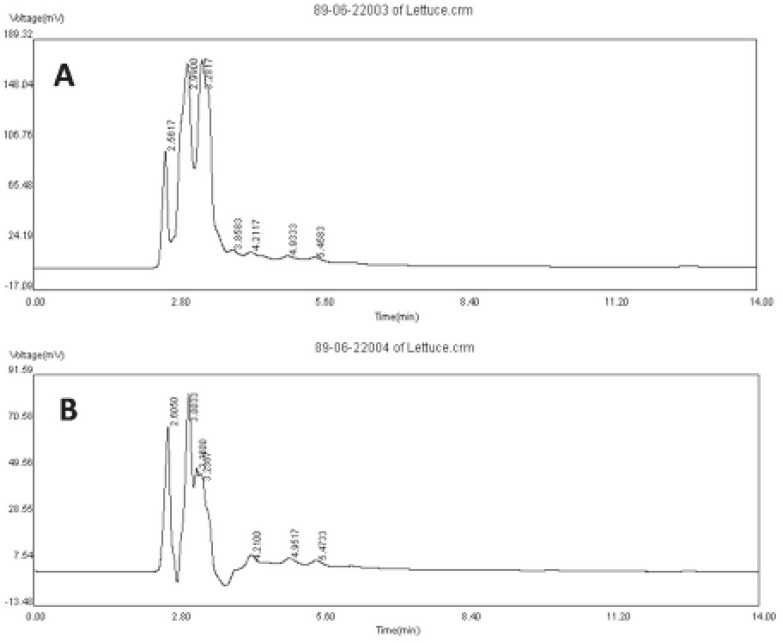
HPLC fingerprints of hydro-alcoholic extract (A) and *n*-butanol fraction (B) of *Lactuca sativum*. Chromatogram detected by UV at 330 nm.

The HPLC fingerprint is a fast method to evaluate the quality of extracts and to provide information about the proportion of the main constituents. In the fingerprints of HAE and NBF of lettuce, there were seven common peaks within retention time range of 2-6 min (Figure 3). 

In both profiles, peak 4 showed the greatest difference and was more prominent in NBF fingerprint. Therefore, its constituent(s) may be responsible for sleep enhancing effect of the extract. In future studies, it would be interesting to elucidate if the constituent(s) are terpenoid agents. 

In conclusion, the present data, for the first time, demonstrate that lettuce potentiates the pentobarbital-induced sleeping behaviors in mice. The sleep prolonging effect was comparable to that of induced by diazepam and accompanied with no neuron toxicity. The main component(s) responsible for the effects are most likely of non-polar agents found in NBF. Isolation of the active compound(s) may yield novel sleep-prolonging agents. 
